# Editorial: Non-Coding RNAs as Mediators of the Activity of Natural Compounds

**DOI:** 10.3389/fphar.2021.751956

**Published:** 2021-08-19

**Authors:** Egeria Scoditti, Alessio Naccarati, Sara Carpi, Beatrice Polini, Sherif S. Ebada, Paola Nieri

**Affiliations:** ^1^ National Research Council (CNR) Institute of Clinical Physiology (IFC), Lecce, Italy; ^2^ Italian Institute for Genomic Medicine (IIGM), c/o IRCCS Candiolo, Torino, Italy; ^3^ Candiolo Cancer Institute, FPO-IRCCS, Torino, Italy; ^4^ Department of Pharmacy, University of Pisa, Pisa, Italy; ^5^ NEST, Istituto Nanoscienze-CNR and Scuola Normale Superiore, Pisa, Italy; ^6^ Department of Pharmacognosy, Faculty of Pharmacy, Ain Shams University, Cairo, Egypt; ^7^ Department of Pharmacognosy, Faculty of Pharmacy, Sinai University, Ismailia, Egypt

**Keywords:** non-coding RNA, micro-RNA, lnc-RNA, medicinal plant, diet, XenomiR

Only a minimal percentage (2%) of DNA is transcribed to messenger RNA (mRNA) and thus to proteins, while about 75% is transcribed in non-coding RNAs (ncRNAs) that are relevant in regulating cell function through their epigenetic role (Statello et al., 2021). In the last decades, among small ncRNAs (<200 nucleotides), microRNAs (miRNA) have been mostly investigated, but an interest has also grown on long ncRNAs (lncRNAs, >200 nucleotides), circular RNAs (circRNAs) and other RNA molecules, revealing their interesting ability in transcriptionally and post-transcriptionally regulating gene expression and thus in controlling human health and response to the environment (diet, xenobiotics, stress, etc.) (Miguel et al., 2020). The investigation of ncRNAs role in the mechanism(s) of action of animal- or plant-derived bioactive natural compounds (NCs) is important to deeply explore and understand their efficacy as preventive and/or therapeutic agents, together with their possible undesired effects and interaction with other chemical agents. Literature regarding miRNA-modulation by dietary factors and especially NCs as a mechanism mediating their improvement of cancer therapy and prevention (Biersack, 2016; Peng et al., 2019; Zou et al., 2021) or contrasting other chronic diseases, such as obesity (Del Carmen Martinez-Jimenez et al., 2018) and cardiovascular diseases (Kura et al., 2019), was recently reviewed.

In this context, the research articles and reviews collected in this Research Topic give further support to the role of ncRNAs in the NCs activity, confirming that the study of epigenetic mechanisms is relevant to comprehend the response of human body to dietary factors and NCs and evaluate their role as potential therapeutic agents.

The review by Sabo et al. discusses recent advancements regarding the role played by miRNAs and lncRNAs in mediating the effects of plant-derived NCs against different cancers. The available data retrieved from studies in cancer cell lines and tumor bearing mice models point to a beneficial inhibition by main classes and subclasses of natural compounds of different specific cancer-related pathways and processes, including growth, invasion, migration, energetic metabolism, angiogenesis, inflammation, and metastasis. Many of these effects are accounted for by the modulation of the expression levels (often dysregulated) of different miRNAs and less frequently lncRNAs and their downstream targets, by acting as tumor-suppressors, oncogenic, and/or implicated in chemoresistance and chemosensitivity. As highlighted in the review, much research is still warranted in the basic lncRNA-mediated mechanisms and clinical efficacy, but the field of NCs-based therapeutics appears as one of the most valuable and promising for cancer treatments.

In line with this role, the paper by Carpi et al. adds an interesting finding regarding Oleacein, an abundant secoiridoid present in *Olea europeae* L. tree and extra-virgin olive oil (EVOO) and thus a relevant component in the Mediterranean diet. In particular, Oleacein has been showed the ability to inhibit melanoma cell (501Mel) proliferation, acting on specific miRNAs (miR-193a-3p, miR-193a-5p, miR-34a-5p, and miR-16-5p miR-214-3p) and their downstream mRNA targets coding for proteins of the Bcl-2 family and mTOR pathway, contrasting cell survival, proliferation and apoptosis resistance. This study therefore supports the role of miRNA modulation in the possible cancer prevention activity of EVOO phenol compounds.

ncRNAs can be useful also as biomarkers in blood and other biological fluids (Wen et al., 2020), which may be investigated to reveal efficacy of NCs. Ferrero et al. describes an interesting nutri-epigenomic study on 120 healthy subjects, which demonstrates that dietary intake may affect the expression levels of several circulating miRNAs. Twenty-three NCs of the classes of lipids, micro-elements, and vitamins were considered for correlation with miRNA expression. The Authors identified, by small RNA-sequencing of plasma samples, 78 NC-miRNA relations consistent among three different dietary groups (vegans, vegetarians, and omnivores). Among all the analysed NCs, sodium, cholesterol, vitamin D, and vitamin E displayed the highest number of correlations with miRNA expression profiles. These findings showed that nutrient composition of habitual diets may influence circulating miRNA profiles, highlighting the relevance of nutri-epigenomic research for further investigations on the molecular impact of diets with potential (patho) physiological outcomes.

The LncRNAs p50-associated cyclooxygenase (COX)-2 extragenic RNA (PACER) and long intergenic non-coding RNA (lincRNA-p21) are implicated in inflammation and cell response to DNA damage *via* exerting anti-inflammatory effects and/or inducing cell cycle arrest and apoptosis in various cell types. Therefore, they are pointed to as potential targets for combating serious inflammatory conditions and/or infections. Tamgue et al. studied the anti-bacterial activity of triptolide, a diterpene triepoxide from a Chinese medicinal plant widely used to treat various inflammatory disorders. *Mycobacterium tuberculosis* (Mtb) was selected as a test microorganism due to its role in inhibiting host lncRNAs expressions to escape host’s defense mechanisms, such as the induction of pro-inflammatory cytokines/enzymes including interleukin (IL)-6 and prostaglandin synthase-2 (Ptgs-2 also known as COX-2), and apoptosis mediated by lncRNA-PACER and lincRNA-p21, respectively. Their work plan was directed toward assessing lncRNAs as mediators of triptolide’s activities on macrophages, which are as an intriguing component in the host immune response. The obtained results revealed an unprecedented evidence that triptolide modulates the expression of both lncRNAs along with their target genes IL-6 and Ptgs-2 in both resting and Mtb-infected human macrophages. This suggests a possible linkage between lncRNAs expression, induction of pro-inflammatory activities in macrophages and the intracellular Mtb-killing activity of triptolide.

If the involvement of endogenous ncRNAs in the activity exerted by NCs is increasingly recognised, the ability of diet-derived ncRNAs, particularly miRNAs, named xeno-miRs, to affect gene expression of host cells is still debated (Zhao et al., 2018). Nevertheless, a role for plant and bacterial miRNAs in inter-kingdoms communication is supported by several evidences (Zhao et al., 2018). The Topic paper by Minutolo et al. confirmed this role of xeno-miRs, reporting the effects of plant-enriched purified extract miRNAs (p-miRs) from *Moringa oleifera* seeds (MO) on the immune response against HIV infection. The peripheral blood mononuclear cells from HIV-positive patients treated with MO-derived p-miRs showed an induced Fas- and Bcl2-mediated apoptosis, a reduced TNF-α expression and a modified pattern of differentiated CD4 T-lymphocytes. Altogether, these modulations by *Moringa oleifera* Lam miRNAs resulted in reduced replication of HIV-infection.

In conclusion, this Research Topic supports the relevance of ncRNAs in the molecular mechanisms underlying the biological response of human body to NCs, both in health and disease conditions. A particular feature linking NCs and ncRNAs is the possibility that NC-derived xeno-ncRNAs may represent bioactive NCs themselves, thus paving the way to potential new preventive or therapeutic opportunities.
